# *IL-12* based gene therapy in veterinary medicine

**DOI:** 10.1186/1479-5876-10-234

**Published:** 2012-11-21

**Authors:** Darja Pavlin, Maja Cemazar, Gregor Sersa, Natasa Tozon

**Affiliations:** 1 University of Ljubljana, Veterinary Faculty, Small Animal Clinic, Cesta v Mestni log 47, Ljubljana, 1000, Slovenia; 2 Institute of Oncology Ljubljana, Department for Experimental Oncology, Zaloska 2, Ljubljana, 1000, Slovenia

**Keywords:** Interleukin-12, Interferon-γ, Gene therapy, Dogs, Cats, Horses, Plasmid, Electroporation

## Abstract

The use of large animals as an experimental model for novel treatment techniques has many advantages over the use of laboratory animals, so veterinary medicine is becoming an increasingly important translational bridge between preclinical studies and human medicine. The results of preclinical studies show that gene therapy with therapeutic gene encoding interleukin-12 (IL-12) displays pronounced antitumor effects in various tumor models. A number of different studies employing this therapeutic plasmid, delivered by either viral or non-viral methods, have also been undertaken in veterinary oncology. In cats, adenoviral delivery into soft tissue sarcomas has been employed. In horses, naked plasmid DNA has been delivered by direct intratumoral injection into nodules of metastatic melanoma. In dogs, various types of tumors have been treated with either local or systemic *IL-12* electrogene therapy. The results of these studies show that *IL-12* based gene therapy elicits a good antitumor effect on spontaneously occurring tumors in large animals, while being safe and well tolerated by the animals. Hopefully, such results will lead to further investigation of this therapy in veterinary medicine and successful translation into human clinical trials.

## Background

The concept of gene therapy involves the transfer of genetic material into target cells to overcome a genetic defect or to provide a protective or corrective function with the goal of curing a disease or improving the clinical status of a patient [1]. In the case of genetic disease caused by mutation in a specific gene, the therapeutic effect of gene therapy is usually achieved by the delivery of a functional gene into target cells or tissue. Exogenous gene delivery can also be used as an immune therapy for the treatment of genetic or non-genetic disorders. The first human gene therapy trial was conducted in the USA in 1990 for the treatment of severe immunodeficiency due to adenosine deaminase deficiency [2]. One year later, the first clinical trial was performed employing gene therapy in the treatment of cancer (melanoma) [3]. Since then, over 1500 clinical studies have been initiated for various indications, the majority, over 2/3, in cancer therapy.

Current preclinical and clinical studies employing gene therapy of cancer show that this treatment approach can vastly improve the therapeutic outcome of oncologic patients as either single or adjuvant therapy. Unfortunately, however, the initial human clinical studies employing gene therapy also revealed important safety concerns [4,5]. Although laboratory animals have been invaluable for research of gene therapy, the results of such studies cannot be directly translated to human patients. Veterinary medicine is thus becoming an increasingly important translational bridge from *in vitro* and preclinical studies to human medicine. Studies on large animal models can complement research on laboratory animals and facilitate more accurate translation from the preclinical to the clinical level, especially in studies that are not possible in human patients for various reasons.

The use of large animals, mainly dogs, cats, horses and cattle, as an experimental model for novel treatment techniques, has many advantages over the use of laboratory animals [6]. These species, especially companion animals, and people share the same living environment and are therefore subjected to similar environmental risk factors for the development of certain diseases. They share many anatomic and physiologic similarities with humans and can display similar clinical signs as affected humans, indicating that comparable genetic mechanisms are responsible for a particular disease in these species. Dogs, cats and horses have much longer life spans than laboratory animals and can therefore naturally reach the age commonly associated with the highest risk for cancer, which makes them especially valuable as natural models for research in oncology.

Oncologic clinical studies in pets can therefore be performed on spontaneously occurring tumors, which have different characteristics than experimentally induced tumors, including inter-individual and intra-tumoral heterogeneity, the development of recurrent or resistant disease, and metastasis to relevant distant sites. Due to pets’ considerably longer lifespans compared to laboratory rodents, possible long-term side effects and other limitations to novel experimental therapies can be detected more accurately. All that, in combination with the lack of established gold-standard veterinary treatment protocols for many diseases, but especially cancer, provides the opportunity for the early and humane evaluation of various new therapies, particularly gene therapy. Experiments on large animal models therefore provide proof of principle and help discern the potential efficacy and safety of gene transfer, which cannot be accurately determined on laboratory animals [6,7].

### IL-12 based gene therapy

Interleukin-12 (IL-12) was discovered independently by two different research groups, Trinchieri and colleagues as a "natural killer-stimulating factor" in 1989 [8] and by Gately and colleagues in 1990 as a "cytotoxic lymphocyte maturation factor" [9]. It has been identified as a heterodymeric protein, composed of two subunits, α-chain with a molecular mass of 35 kDa, also known as p35 or IL-12α, and β-chain with molecular mass of 40 kDa, also known as p40 or IL-12 β**,** which are covalently linked by a disulfide bridge. Shortly after the discovery of IL-12, genes for IL-12 in various species were cloned, which stimulated further investigations relating to the therapeutic potential of this newly discovered cytokine [10].

From the beginning, three activities of IL-12 were already recognized: induction of IFN-γ response, amplification of NK cell-mediated cytotoxicity and mitogenic response of T cells [8]. Based on these biological actions, it was predicted that this cytokine is required for resistance to bacterial and intracellular parasites, as well as for the establishment of organ-specific autoimmunity [11] and it was considered that it shows possible therapeutic potential for the treatment of diseases that would favorably respond to its immunomodulatory actions. With the additional discovery of its anti-angiogenic effects [12], IL-12 became one of the most promising cytokines for the treatment of malignant disease.

A model of mechanisms involved in the antitumor effects of IL-12 predicts that IL-12 directly activates cells of the adaptive (CD4+ and CD8+ T cells) and innate arms of immunity by helping to prime T cells, increasing their survival, enhancing T cell and NK cell effector functions, as well as promoting the induction of INF-γ secretion. IFN-γ in turn acts directly on cell components within the tumor, by enhancing the recognition of tumor cells through MHC class I processing and presentation and by modifications of the extracellular matrix, which results in reduced angiogenesis and tumor invasion. The end result of these actions is delay of tumor growth and, ultimately, eradication of the tumor [13].

In the first preclinical studies on the antitumor effectiveness of IL-12, recombinant IL-12 (rIL-12) protein was used. Despite showing a significant effect in these studies, the success was not adequately translated into the clinical setting. Systemic application of recombinant IL-12 resulted in unexpected severe toxicity, even at doses as low as 1 μg/kg/day [14], which proved to be a major obstacle to further progression of rIL-12 into clinical practice. The most common adverse effects of rIL-12 therapy include elevated body temperature, headache, weakness and gastrointestinal toxicity (stomatitis, nausea and vomiting). Laboratory changes associated with rIL-12 toxicity include anemia, neutropenia, lymphopenia, hypoglycemia, thrombocytopenia, hypoalbuminemia and liver function test abnormalities. In phase II human clinical trials, systemic rIL-12 therapy even resulted in the death of treated patients [15]. New strategies for introducing IL-12 were therefore studied, one of them being delivery of the gene encoding IL-12, instead of application of recombinant protein. The gene delivery systems for *IL-12* include various viral vectors, *e.g.,* adeno- [16-18], pox- [19] or Semliki Forest virus [20], and non-viral techniques, *e.g.,* transfer of naked plasmid DNA alone [21], gene gun technique [22], electroporation [23-25] and other non-viral methods [26].

### *IL-12* based gene therapy in cats

In cats, most gene therapy trials presently focus on cancer, mainly fibrosarcoma, but also on neurological diseases, various inherited diseases (*e.g.,* mucopolisaharidosis VI) and heart disease [27-32].

To our knowledge, there is currently only one report concerning *IL-12* based gene therapy in cats, in which viral vector was used in 13 cats with spontaneous soft tissue sarcomas, with an additional 16 cats included in the feasibility study with viral construct expressing green fluorescent protein alone or in combination with murine *IL-12*[33] (Table 1). Feline soft tissue sarcomas are a relatively infrequent, but clinically very challenging occurrence, with an estimated incidence between 0.5 – 1 per 10,000 cats [34]. Despite a variety of different treatment modalities that are currently available for the treatment of this disease, the prognosis of affected cats is poor, with reported recurrence rates from 40 to 70%, even with a multimodality approach, combining surgery, radiotherapy and chemotherapy [35,36]. New therapeutic approaches for the treatment of feline soft tissue sarcoma are therefore being investigated, including gene therapy, in which mainly *IL-2* has been used, delivered using magnetofection [37], xenogeneic cells [38] or poxviral vector [39]. In addition to *IL-2*, therapeutic genes encoding *GM-CSF*[27,37] and *IFN-γ*[37] and viral suicide gene therapy [40] have also been used.

**Table 1:** **Summary of*IL-12*gene therapy studies in veterinary medicine**

	**No. and type of animals included in the study**	**Study design**	**Type of treated tumors**	**Type of gene delivery**	**Route of gene delivery**	**Type of therapeutic IL-12 gene**	**Treatment outcome**	**Ref**
1	16 cats (GFP ± *mIL-12*) + 13 cats (*fIL-12*)	phase I dose escalation study on naturally occurring tumors	soft tissue sarcomas	viral delivery (adenovirus controlled by heat-inducible promotor)	i.tu.	murine feline	systemic toxicity at high adenoviral doses high expression of IL-12 in all tumors IFN-γ intratumoral expression detected only with high doses side effects correlated with IFN-γ expression	[33]
2	7 horses	phase I/II study on naturally occurring tumors	metastatic melanoma	direct plasmid injection	i.tu.	human	41% mean reduction of tumor size after single plasmid injection (11/12 treated tumors) CR after 3 plasmid injections in 1/12 tumors only short response (regrowth 11/12 tumors) histological change of treated tumors no side effects	[46]
3	8 horses	phase II/III placebo-controlled study on naturally occurring tumors	metastatic melanoma	direct plasmid injection	i.tu.	equine	regression in tumor size, with mean volume of treated tumors decreasing to approximately 80% of baseline value side effect: local peritumoral oedema of smaller treated lesions	[48]
4	7 horses	pharmacokinetics study	metastatic melanoma	direct plasmid injection	i.tu.	equine	plasmid enters peripheral blood 10 minutes after intratumoral DNA application and is present up to 36 hours post injection, with peak concentration at 30 minutes intratumoral expression of IFN-γ was detectable in all melanoma samples with high interindividual variability	[47]
5	6 dogs	dose escalating study on experimentally induced tumors	transmissible venereal tumors	EGT	i.tu.	human	statistically significant growth delay of treated tumors CR in all of the treated tumors systemic release of IL-12 and/or IFN-γ antitumor effect on distant untreated tumors	[66]
6	8 dogs	phase I/II study on naturally occurring tumors	mast cell tumors	EGT	i.tu.	human	50% median reduction of tumor volumes (ranging from 15 – 83%) systemic release of IL-12 and/or IFN-γ change in histological structure of treated tumors	[56]
7	7 dogs	phase I feasibility and safety study	N/A	EGT	i.m.	human	systemic release of IL-12 (1/6 dogs) induction of IFN-γ response (3/6 dogs) no detectable side effects	[76]
8	6 dogs	phase I/II study on naturally occurring tumors	different types of tumors	EGT	i.m.	human	systemic release of IL-12 and/or IFN/γ in 4/6 animals prolongation of patients' life	[65]
9	N/A	description of ECGT protocol/case report	head and neck tumors	ECGT (*IL12* + BLM)	i.tu.	N/A	report on eradication of two tumors (the same two patients are also presented in the study under no. 10)	[72]
10	6 dogs	phase I/II study on naturally occurring tumors	different types of highly malignant tumors	ECGT (*IL-12* + BLM)	i.tu.	feline	CR 3/6 dogs PR 3/6 dogs	[57]

Ref: reference; GFP: green fluorescent protein; mIL-12: murine IL-12; fIL-12: feline IL-12; EGT: electrogene therapy; ECGT: electrochemogene therapy; i.tu. intratumoral; i.m.: intramuscular; BLM: bleomicyn; CR: complete response; PR: partial response.

Siddiqui and colleagues performed a phase I dose escalation study using adenoviral vector with feline *IL-12* gene controlled by heat-inducible promoter, as adjuvant therapy to fractionated radiotherapy [33]. The cats underwent 16 fractions of radiation therapy over 22 days, followed by gene therapy performed as intratumoral injection of viral vector 3–5 days after completion of the radiotherapy protocol. Three cohorts of cats were studied at dose levels of 10^9^, 10^10^ and 4 × 10^10^ pfu of adenovirus per tumor. Twenty-four hours later, treated tumor nodules were heated to approximately 40–44°C, which allowed spatial and temporal control of *IL-12* expression.

Since this study was designed as a phase I study, the main focus was on establishing the maximum safe dose of the viral construct and the detection of possible side effects in the light of well recognized side effects of adenoviral gene therapy (immunogenicity) and IL-12 therapy. No treatment-related mortality was seen, although serious side effects were detected in the cats that received the highest dose of viral construct. These included lack of appetite, lethargy, pulmonary edema and hepatic and hematologic toxicities, which even required intensive care support for up to 2 weeks. IL-12 and IFN-γ mRNA levels were measured using RT-PCR in the treated tumors 24–48 hours after the induction of hyperthermia, revealing a consistently high IL-12 mRNA relative fold increase over the baseline in all tumor samples taken at the 24-hour time point. A significant increase of IFN-γ mRNA was detected in only 5/13 cats (mainly in the group receiving the highest dose of viral vector) and the magnitude of the relative fold increase was much lower compared to IL-12. Cats in which high levels of IFN-γ were detected showed clinical signs of systemic toxicity. Since IFN-γ has been implicated as the cytokine directly responsible for IL-12 toxicity [15,41], it can be postulated that, in these cats, locally produced IL-12 induced an IFN-γ response, resulting in systemic shedding of IFN-γ, leading to systemic toxicity.

### *IL-12* based gene therapy in horses

The horse is most commonly used as a large animal model for osteoarthritis and melanoma, which features similar histologic and immunohistologic characteristics as melanoma in humans [42]. Grey horses develop metastatic melanoma spontaneously and are especially suitable as a large animal model for melanoma since a genetic predisposition for this disease is recognized [43] and distant metastases occur spontaneously to similar organs as in the human disease. Conventional oncologic therapies, such as surgery, cryosurgery or local chemotherapy, have only a limited antitumor effect [44]. In horses, gene therapy of this disease has been attempted employing suicide gene therapy [45] or therapeutic genes encoding *IL-12* and *IL-18*[46-48].

As an alternative treatment modality to the conventional therapeutic approach, several studies have used direct intratumoral injection of *IL-12* plasmid without any additional physical or chemical delivery method [46-48] (Table 1). Various publications have described different aspects of such therapy in altogether 22 grey horses with multiple tumor nodules. In the first study [46], plasmid encoding human *IL-12* was used, injected intratumorally one to four times in altogether 12 nodules in 7 horses. This therapy resulted in tumor regression, with a 41% mean reduction of tumor size after a single plasmid injection and in one nodule a complete response was even achieved with three consecutive plasmid injections. The lowest tumor volumes were seen from day 10 onward until day 99 after therapy and, after reaching the smallest volume, tumor nodules slowly regrew. The local response to the therapy was therefore only short term, but the tumors responded again to repetition of the therapy, which leads to the conclusion that repetitive applications of intralesional IL-12 gene therapy at 30-days intervals would be needed to achieve a more pronounced clinical effect. Local response to the therapy was also confirmed with histological evaluation of the treated tumors, which revealed significant peritumoral infiltration with both CD4+ and CD8+ lymphocytes, which was not observed in untreated tumors. However, the therapy did not result in systemic shedding of the transgene product in the treated animals, since no detectable concentrations of human IL-12 were found in blood samples taken at different time points from one to 30 days after plasmid injection. Results of this study proving safety and efficacy also provided basis for a subsequent human clinical phase I study using the same treatment approach in late-stage malignant melanoma patients [49].

A decade later, the same research group performed another comprehensive study of the pharmacokinetics and antitumor effects of intratumoral injection of *IL-12* in metastatic melanoma in grey horses, using equine instead of human *IL-12* construct [47]. A thorough evaluation of the pharmacokinetics was performed on 7 horses, in which multiple blood samples and biopsies at the site of injection were taken at 13 time points immediately before and up to 14 days after intratumoral plasmid injection. Plasmid DNA and mRNA was isolated from the blood samples and quantified using the RT-PCR method. It was established that plasmid entered the peripheral blood as soon as 10 minutes after intratumoral DNA application and was present up to 36 hours post injection, with a peak concentration at 30 minutes and an exponential decrease thereafter. Intratumoral expression of IFN-γ was detectable in all melanoma samples, with high inter-individual variability. IFN-γ expression decreased with time, but much more slowly compared to the clearance of IL-12 plasmid from peripheral blood, with IFN-γ remaining significantly elevated above the baseline value at the time of the last sampling (*i.e.,* 14 days after intratumoral injection of the plasmid).

The antitumor effect of the same therapeutic plasmid was evaluated in a double-blind placebo-controlled study on 8 horses with 22 melanoma nodules [48]. Each tumor was injected with a cumulative dose of approximately 1.5 mg of equine IL-12 plasmid, divided into 6 consecutive applications. *IL-12* gene therapy elicited regression in tumor size, with the mean volume of treated tumors decreasing to approximately 80% of the baseline value. Histologic examination of biopsies in a smaller number of treated horses revealed similar changes as in the earlier study, *i.e.,* intra- or peritumoral mild perivascular lymphocytic infiltrations.

The safety aspect of *IL-12* gene therapy was addressed in all three studies on grey horses, with regular clinical examinations of the treated animals, as well as hematologic and biochemical examinations of blood. Side effects included only local peritumoral oedema of smaller treated lesions (< 1 cm in diameter), which lasted from 1 to 3 days after plasmid injection. No systemic side effects were detected, revealed either as a change in the clinical status of animals or laboratory abnormalities, indicating that such a therapeutic approach can be considered an effective as well as safe procedure in grey horses, with only minimal transient local side effects.

### *IL-12* based gene therapy in dogs

The most commonly used large animal in gene therapy research is the dog. An important advantage of the use of dogs compared to laboratory rodents or other pet animals is the similarity of canine and human immune systems [50]. Furthermore, successful sequencing of the canine genome [51] has led to characterization of a variety of genetic disorders in dogs, such as hemophilia, mucopolisaharidosis VII, and various cardiovascular diseases and cancers. A number of clinical studies have also been initiated for the treatment of canine cancers, using different therapeutic genes, such as Fas ligand, bacterial superantigens and cytokines, including GM-CFS, IL-2 and IL-12 [52-58]. Another palliative approach to tumor-bearing dogs employed gene therapy with gene encoding growth hormone releasing hormone in order to ameliorate tumor cachexia and improve the general clinical status of patients [59].

The majority of *IL-12* gene therapy studies in large animals have been performed on dogs, all of them utilizing electrogene therapy of various types of tumors, either as a single therapeutic approach or as adjuvant therapy to other treatment methods (Table 1). Electrogene therapy (EGT) is a procedure in which electroporation is used for the delivery of various therapeutic genes into target tissue. Electroporation, *i.e*., increasing the permeability of the cell membrane using the application of controlled electric pulses, has been used as a physical method for increased intracellular delivery of a range of different molecules since the beginning of the 1980s [60]. Electroporation-based gene delivery has been used *in vivo* since the beginning of the 1990s [61,62] and in the last quarter of the century, transgenic DNA was successfully introduced in a vast number of different tissues in a variety of different species [63].

The antitumor effectiveness of *IL-12* EGT has already been established in a number of different experimental tumor models, including melanoma, lymphoma, different carcinomas and sarcoma [64]. The most pronounced effect of such therapy was growth delay or even complete long-term eradication of treated tumors. Furthermore, it generated long-term resistance to the development of new tumors, reduced the number of lung metastases and consequently prolonged the life of the treated animals compared to control groups [64].

Based on the results of preclinical studies, the first human clinical study using intratumoral *IL-12* EGT on 24 patients with cutaneous metastatic melanoma was performed [65]. Three EGT procedures were performed on days 1, 5 and 8, resulting in a good local clinical response of the treated tumors, along with a systemic antitumor effect on untreated distant nodules in 53% of patients.

### Intratumoral *IL-12* EGT in dogs

In dogs, *IL-12* EGT has been investigated on a number of different induced and spontaneously occurring tumors [57,58,66,67]. In these studies, either human or feline *IL-12* was used as a therapeutic gene due to the non-availability of canine *IL-12* and high homology between canine and these two cytokines [68]. Based on amino acid sequence analysis, canine IL-12 shares an approximately 90% genetic identity with both human and feline IL-12. Furthermore, these two types of IL-12 activate the proliferation of canine peripheral blood mononuclear cells (PBMC) *in vitro* and trigger a number of immune responses in canine PBMC [58,69], which has led to speculation that they also have an *in vivo* biologic effect in dogs. This assumption was confirmed for both cytokines, based on the fact that they displayed a good antitumor effect in various canine tumors, eliciting similar biologic changes in tumors as in *in vitro* and preclinical studies.

Chuang and colleagues employed *IL-12* EGT in the treatment of experimentally transplanted transmissible venereal tumors (TVT) in 6 experimental beagle dogs [67]. Tumors were established by subcutaneous injection of 10^8^ canine TVT cells, which originate from spontaneous TVT tumor [70] at 8 sites on the back and treated when the diameter of the tumors reached 1–2 cm. One mg of plasmid DNA was injected intratumorally into 16 nodules, followed by electroporation. This treatment showed remarkable antitumor efficacy, with significant growth delay in all treated tumors, achieving long-term complete regression, without tumor regrowth in the one-year observation period. Furthermore, in a dose-escalating study, even the lowest injected dose (*i.e.,* 0.1 mg of plasmid) elicited significant growth delay in the treated tumors. *IL-12* EGT also had a pronounced systemic effect on distant untreated tumors and induced long-term resistance to tumor regrowth after re-challenge with subcutaneous injection of the same tumor cells. Similar systemic effects have also been shown in experimental tumor models, *e.g*., fibrosarcoma [71] and in a human clinical study [65].

**Figure 1:**
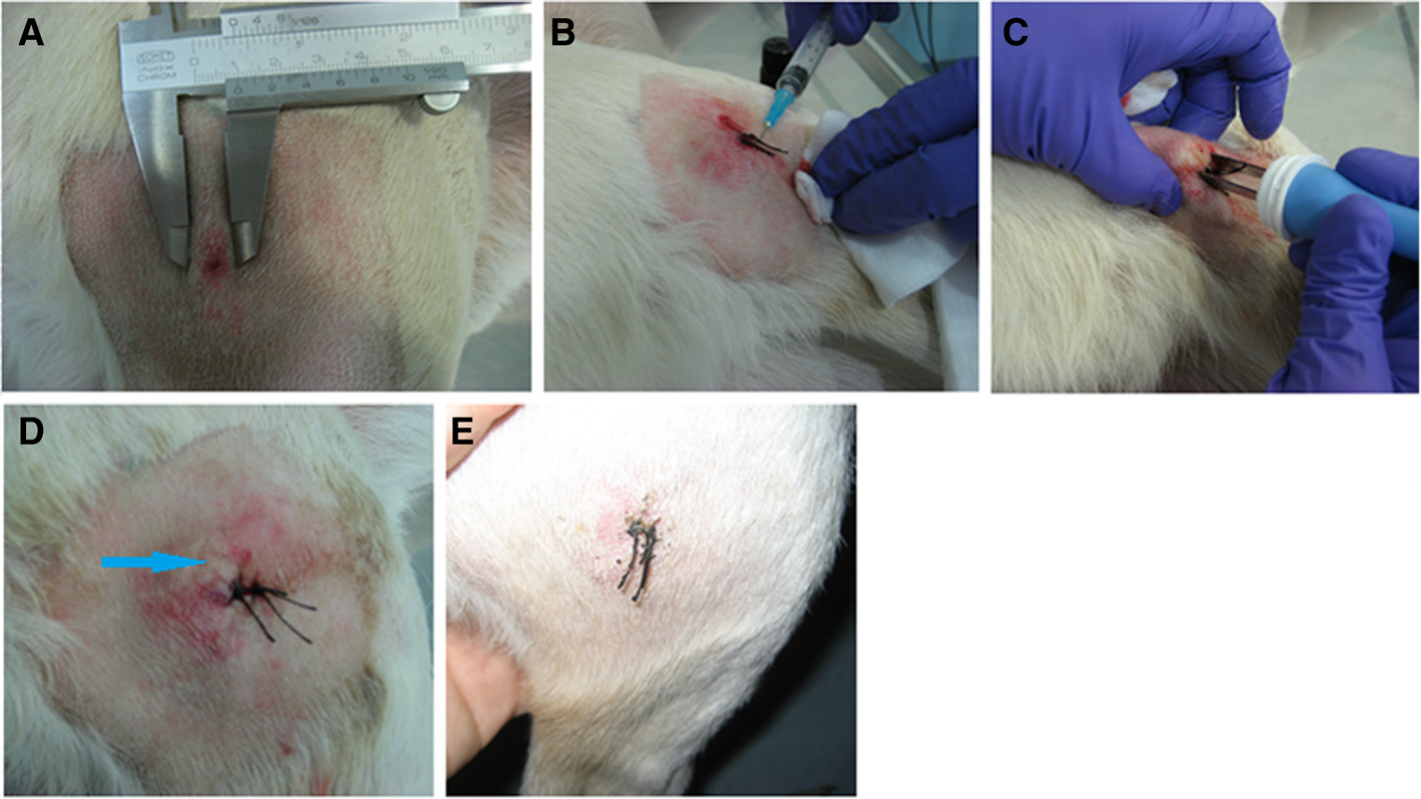
**Intratumoral EGT procedure in a dog with a mast cell tumor in the gluteal region.A**: tumor before the procedure; **B**: intratumoral injection of IL-12 plasmid; **C**: delivery of electric pulses using plate electrodes with electric pulse generator Cliniporator® (Igea s.r.l., Italy); **D**: nodule immediately after the procedure. A marked paleness of the electroporated area compared to the surrounding tissue can be seen (arrow), due to transient reduction of the blood flow to the tissue, caused by occlusion of blood vessels during electroporation; **E**: tumor 10 days after therapy.

A clinical study in naturally occurring mast cell tumors in dogs was performed at our institution [57] (Figure 1). It was conducted on 11 tumor nodules from 8 dogs with intratumoral *IL-12* EGT, displaying a good local antitumor effect and systemic release of transgene products. The reduction in tumor size was not as pronounced as in experimentally induced TVTs, since no complete response was achieved, but the size of treated nodules decreased in the range of 15 – 83% (median 50%) from the initial tumor volume. Additionally, a change in the histological structure of the treated tumors was seen, with a reduction in the number of malignant mast cells and diffuse infiltration of treated tumors with lymphocytes and plasma cells, as well as degranulation of the remaining mast cells. Similar histological changes are characteristic of plasmid-based *IL-12* gene therapy, in which one of the most prominent histologic changes found is intra- and peritumoral lymphocytic infiltration [46,48,65,72]. The pivotal role of lymphocytic infiltration after intratumoral *IL-12* EGT has been demonstrated in preclinical studies, in which no antitumor effect of such therapy was achieved in athymic mice deficient in T cells, compared to immunocompetent mice [71,73]. However, another study provided evidence to the contrary, since a significant regression of human melanomas in nude athymic mice was achieved with repeated intratumoral injections of *IL-12* plasmid, indicating that antitumor effect of IL-12 probably also arises from other modulations of specific and unspecific immune response mechanisms [74].

**Figure 2:**
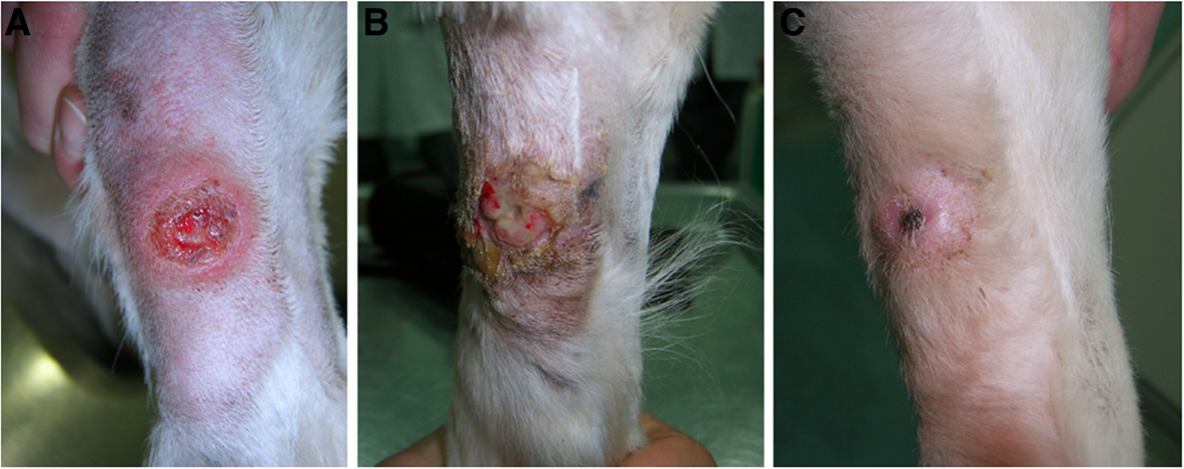
**Mast cell tumor on the front leg of a dog, treated with a combination of ECT with cisplatin and*IL-12*EGT as part of an ongoing clinical study at our institution.A**: a large ulcerated tumor nodule before therapy, **B**: one week after the procedure, massive necrosis of the treated area can be seen; **C**: one month after the procedure, a cytologically confirmed complete response was achieved.

An additional two publications have described the implementation of a combination of electrochemotherapy (ECT) and intratumoral EGT (*i.e*., electrochemogene therapy (ECGT)) [58,75] (Figure 2). In ECT, electroporation is used for intracellular delivery of various chemotherapeutics (mainly bleomycin and cisplatin) for potentiating the antitumor effect of these drugs [76,77]. Since both ECT and EGT are based on the use of the same physical phenomenon, they are especially suitable for combined use. Reed and colleagues [58] combined intratumoral application of approximately 0.5 IU of bleomycin and 150 μg of IL-12 plasmid per cm^2^ of tumor in 6 dogs with naturally occurring tumors, mainly carcinomas and sarcomas. The majority of them were high-grade tumors with a statistically poor prognosis (*e.g*., histiocytic sarcoma, fibrosarcoma and malignant melanoma with high mitotic index). A clinical response to ECGT was observed, with minimal side effects regardless of the tumor type. In three of the 6 treated dogs (two with oral squamous cell carcinoma and one with acanthomatous ameloblastoma), a complete response was achieved and the remaining three (one of each: histiocytic sarcoma, metastatic melanoma and fibrosarcoma) had a partial response to treatment. Eradication of the superficial treated nodules was most probably predominantly due to previously well described cytoreductive effects of ECT as the bleomycin and plasmid DNA were injected together. However the bystander effect on bone lysis repair in invasive tumors could be at least partially explained by distant effects of IL-12 from the EGT part of the treatment.

Data on intratumoral cytokine production after local intratumoral *IL-12* EGT is available for preclinical studies on experimental animals, although the results are very inconsistent. For example, in experimental melanoma models, intratumoral *IL-12* EGT produced low concentrations of either IL-12 or IFN-γ, with peak levels of both measured cytokines never exceeding 10 pg/mg of tumor tissue [72]. On the other hand, significantly higher concentrations were achieved in *IL-12* EGT of a sarcoma tumor model, with intratumoral cytokine levels as high as 10 ng/mg of tumor tissue for IFN-γ and 50 ng/mg of tumor tissue for IL-12 [70]. Similar information regarding intratumoral concentrations of cytokines after EGT in larger animals is sparse. Intratumoral cytokine production was measured only in a study on experimentally induced TVTs, in which high peak levels (approx. 2.5 ng/mg) were reached 7 days after EGT [67]. Such intratumoral cytokine production is not only important for a direct local antitumor effect of EGT, but more and more data is presented that sufficient intratumoral production of transgene can also result in systemic shedding of encoded product, thus expanding local therapy to the systemic level [70,78]. Systemic shedding of therapeutic molecules is most probably a prerequisite for systemic antitumor effects of gene therapy, for example, the inhibition of growth of untreated tumors at distant sites, an antimetastatic effect and induction of long-term systemic immunity. However, since the data on measurement of systemic release of cytokines are scarce, it cannot be ruled out that the systemic effect can be induced locally. Additionally, the use of feline and human IL-12 in these studies and modest availability of commercial tests makes these studies demanding. Nevertheless, more evidence of systemically detectable levels of IL-12 and/or IFN-γ in animals treated with intratumoral *IL-12* EGT is needed that would clarify this point and would lead to better planning of clinical trials.

Two canine studies featuring intratumoral *IL-12* EGT focused on the detection of systemic release of IL-12 and IFN-γ [57,67]. EGT in both of these studies resulted in systemic release of IL-12 and induction of an IFN-γ response, since both cytokines were detected in blood samples of the treated dogs. In a TVT experimental model, higher IL-12 concentrations were detected (peak levels 145 ± 78 pg/ml) compared to spontaneous mast cell tumors (maximum concentration reached was 12.2 pg/ml). This difference can be explained by much higher doses of plasmid that were used for treatment of the TVTs. In both studies, too, induction of an IFN-γ response was detected, which further verified the biological activity of human IL-12 plasmid in dogs, since one of the most important antitumor actions of IL-12 stems from the induction of IFN-γ production from NK cells. Interestingly, a similar expansion of local gene therapy to the systemic level was also detected in another study on dogs with malignant melanoma, which received an intratumoral injection of lipid-complexed plasmid DNA encoding a bacterial superantigen and one of two cytokines, IL-2 or GM-CSF [53]. The therapy led to high levels of antitumor cytotoxic T-cell activity in the peripheral blood, indicating that local administration of the vector may have produced a systemic effect.

### Intramuscular *IL-12* EGT in dogs

An additional two publications have described a slightly different approach to *IL-12* EGT, *i.e*., intramuscular *IL-12* EGT [66,79]. Our research group performed a feasibility and safety study on electrotransfection of IL-12 plasmid into canine skeletal muscle [78]. The main advantages of using muscle as a target tissue in gene therapy are the high capacity of protein synthesis and post-mitotic status of muscle fibers, which enable long-lasting gene expression, even for more than 1 year [80].

Two different electroporation protocols for efficient muscle transfection were evaluated in six beagle dogs in a dose escalation study using plasmid encoding human IL-12 [78]. Blood samples were collected at different time points after a single intramuscular plasmid delivery, for determination of IL-12 and IFN-γ concentrations and determination of selected haematologic and biochemical parameters. Human IL-12 was detected in the serum of one dog, which received the highest plasmid dose (1 mg) and canine IFN-γ above baseline was detected in three of the six dogs. Hematological and biochemical parameters remained within reference limits throughout the whole observation period (2 months). Based on the results of this study, intramuscular *IL-12* EGT was employed in tumor-bearing dogs [66]. The study was performed on a total of six dogs, three of them with mast cell tumors grade II and III, and the remaining three with pulmonary histiocytic sarcoma, osteosarcoma and mammary adenocarcinoma. Each patient received a single EGT with 1 mg of therapeutic plasmid. In 4/6 treated patients, serum concentrations of IL-12 and/or canine IFN-γ were detected in multiple blood samples (all three dogs with mast cell tumors and the dog with pulmonary histiocytic sarcoma), showing that the therapy elicited systemic release of the encoded transgene and an IFN-γ response. In these four patients, even though the therapy did not have any effect on the volume of measurable tumor nodules, surprisingly long survival times after EGT were achieved, compared to survival times associated with specific tumor types from literature review. For example, the patient with pulmonary histiocytic sarcoma survived more than 8 months after the EGT. According to the literature median survival time for dogs with this type of tumor, treated with chemotherapy, is 3 to 4 months [81,82]. However, the sample size in this preliminary trial was too low and too heterogenic to make statistically significant conclusions regarding effect of intramuscular *IL-12* EGT on patients' survival. The results of this study indicate that intramuscular *IL-12* EGT is a safe procedure in canine cancer patients, which can result in systemic shedding of IL-12 and possibly trigger an IFN-γ response, which could lead to prolonged survival of treated animals.

A particularly important finding of all of these canine studies is that, together with eliciting a good clinical antitumor effect, *IL-12* EGT, applied either intratumorally or intramuscularly, did not cause any significant side effects in the treated animals. In the light of known recombinant IL-12 toxicity, the safety aspect of *IL-12* EGT was very well addressed in all studies. The toxicity of *IL-12* EGT was specifically evaluated in murine melanoma and squamous cell carcinoma tumor models [83,84]. The results of the melanoma study [83] showed that intratumoral EGT did not cause any clinically detectable adverse effects and did not affect laboratory parameters in the treated animals. The only histologically noticeable change was focal inflammation and glomerulosclerosis of the kidneys at a late time point after EGT (around 1 month after the procedure), without any biochemical indicators of diminished kidney function. In the squamous cell carcinoma model [84], mild, dose dependent liver toxicity was noticed, detected histologically and as a transient statistically significant elevation of alanine aminotrasferase. Furthermore, in the first 7 days after therapy, a trend of decreasing total white blood cell counts was observed, which returned to normal values by day 30.

In canine patients, no significant clinical or laboratory changes were observed in any of the published studies. The only reported clinically detectable side effect was 48 hour diarrhea in a dog with malignant melanoma [58], which could not be directly linked to *IL-12* EGT and could be attributed to any number of other causes, since the patient had a host of other health problems. Otherwise, dogs tolerated the procedure very well and did not show any clinical or laboratory indicators of renal, hepatic or systemic toxicity or immunosuppression, which are the most important adverse effects of recombinant IL-12 therapy [15].

## Conclusions

A review of the literature shows that *IL-12* based gene therapy may offer an effective new approach to cancer therapy in veterinary medicine. With various viral and non-viral gene delivery methods, a very good local antitumor effect has been achieved in various tumors of cats, dogs and horses, thus supporting and upgrading the results of a number of preclinical studies featuring IL-12 gene therapy. Studies on large animals have demonstrated that IL-12 based gene therapy is an effective approach, either as local intratumoral gene transfer or as systemic treatment through intramuscular therapeutic gene delivery, targeting either accessible tumor nodules or the disseminated disease. It is a safe and effective therapeutic procedure, which exerts local transgene expression, as well as systemic release of the encoded protein and induction of an IFN-γ response, making it a promising treatment for large animals with spontaneously occurring tumors. Such results will hopefully lead to further investigation of this therapy in veterinary medicine and help in successful translation into human clinical trials.

## Competing interests

The authors declare they have no competing interests.

## Authors’ contributions

DP conducted the background and literature research and drafted the manuscript. MC, GS and NT critically revised the manuscript and provided additional intellectual content. All authors read and approved the final version of the manuscript.
